# Time-to-first exacerbation, adherence, and medical costs among US patients receiving umeclidinium/vilanterol or tiotropium as initial maintenance therapy for chronic obstructive pulmonary disease: a retrospective cohort study

**DOI:** 10.1186/s12890-021-01612-5

**Published:** 2021-07-31

**Authors:** David Slade, Riju Ray, Chad Moretz, Guillaume Germain, François Laliberté, Qin Shen, Mei Sheng Duh, Malena Mahendran, Beth Hahn

**Affiliations:** 1 US Medical Affairs, GSK, Research Triangle Park, NC USA; 2 US Value Evidence & Outcomes, Medical Affairs, GSK, Research Triangle Park, NC USA; 3 Groupe D’Analyse, Ltée, Montreal, QC Canada; 4 Global Value Evidence and Outcomes, GSK, Collegeville, PA USA; 5 Analysis Group, Inc, Boston, MA USA

**Keywords:** Chronic obstructive pulmonary disease, Exacerbation, Initial maintenance therapy, LAMA/LABA, Medication adherence, Medical costs

## Abstract

**Background:**

Adherence to chronic obstructive pulmonary disease (COPD) maintenance medication is important for managing symptoms and exacerbation risk, and is associated with reduced mortality, hospitalizations, and costs. This study compared on-treatment exacerbations, medical costs, and medication adherence in patients with COPD initiating treatment with umeclidinium/vilanterol (UMEC/VI) or tiotropium (TIO).

**Methods:**

This retrospective matched cohort study selected patients from Optum’s de-identified Clinformatics Data Mart database who initiated maintenance treatment with UMEC/VI or TIO between 01/01/2014 and 12/31/2017 (index date defined as the first dispensing). Eligible patients were ≥ 40 years of age and had ≥ 12 months continuous health plan coverage pre- and post-index; ≥ 1 medical claim for COPD pre-index or on the index date; no moderate/severe COPD-related exacerbations on the index date; no asthma diagnosis pre- or post-index; no maintenance medication fills containing inhaled corticosteroids, long-acting β_2_-agonists, or long-acting muscarinic antagonists pre-index or on the index date; and no fills for both UMEC/VI and TIO on the index date. Outcomes included time-to-first (Kaplan–Meier analysis) and rates of on-treatment COPD-related moderate/severe exacerbations, medication adherence (proportion of days covered [PDC] and proportion of adherent patients [PDC ≥ 0.8]), and COPD-related medical costs per patient per month (PPPM). Propensity score matching was used to adjust for potential confounders.

**Results:**

Each cohort included 3929 matched patients. Kaplan–Meier rates of on-treatment COPD-related exacerbations were similar between cohorts (hazard ratio at 12 months; overall: 0.93, moderate: 0.92, severe: 1.07; all *p* > 0.05). UMEC/VI versus TIO initiators had significantly higher adherence (mean PDC: 0.44 vs 0.37; *p* < 0.001; proportion with PDC ≥ 0.8: 22.0% vs 16.4%; *p*< 0.001) and significantly lower mean on-treatment COPD-related total medical costs ($867 vs $1095 PPPM; *p* = 0.028), driven by lower outpatient visit costs.

**Conclusions:**

These findings provide valuable information for physicians considering UMEC/VI or TIO as initial maintenance therapy options for patients with COPD.

**Supplementary Information:**

The online version contains supplementary material available at 10.1186/s12890-021-01612-5.

## Background

In patients with chronic obstructive pulmonary disease (COPD), exacerbations are associated with increased disease burden and higher healthcare costs [1]. As such, reducing the risk of exacerbations is an important goal of maintenance therapy in patients with COPD. The Global Initiative for Chronic Obstructive Lung Disease strategy report recommends initial maintenance therapy (IMT) with either a long-acting muscarinic antagonist (LAMA) or a long-acting β_2_-agonist (LABA) for most patients with COPD [2]. However, single-inhaler dual LAMA/LABA therapy may be considered for appropriate patients, particularly those with more severe symptoms (COPD Assessment Test ≥ 20) or who experience dyspnea or exercise intolerance [2, 3]. The LAMA/LABA combination umeclidinium/vilanterol (UMEC/VI) has demonstrated significantly greater improvements in lung function, symptoms, and health-related quality of life compared with the LAMA tiotropium (TIO) [4–6].

Good adherence to COPD maintenance medication is important in managing symptoms and exacerbation risk and is associated with a reduced risk of mortality and hospitalizations, as well as lower healthcare resource utilization (HCRU) and costs [7–12]. UMEC/VI has been associated with lower lifetime medical costs compared with TIO [13]. However, there is a lack of published studies directly comparing real-world data on the effects of UMEC/VI and TIO on treatable outcomes such as COPD exacerbations and medication adherence.

The aim of the current study was to evaluate and compare on-treatment COPD-related exacerbations, medication adherence, and COPD-related medical costs among patients with COPD who initiated treatment with UMEC/VI compared with TIO.

A plain language summary of this article is included in Additional file 1.

## Methods

### Study design

We used a retrospective matched cohort study design to analyze de-identified medical and pharmacy claims data and enrollment information from Optum’s de-identified Clinformatics Data Mart database, spanning January 1, 2013 to December 31, 2018.

**Fig. 1:**
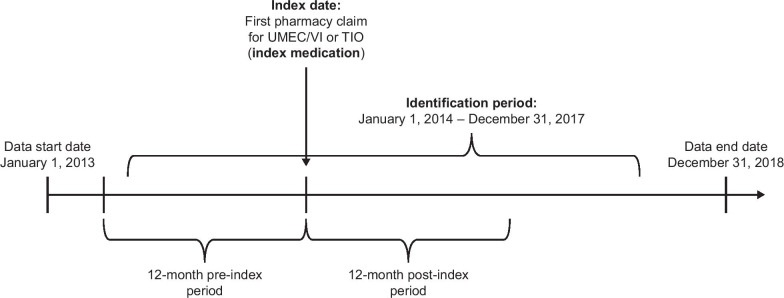
Schematic of study design. UMEC/VI, umeclidinium/vilanterol; TIO, tiotropium

Commercial and Medicare Advantage health plan enrollees who initiated treatment with UMEC/VI or TIO (dry powder inhaler) between January 1, 2014 and December 31, 2017 (identification period; Fig. 1) were identified. The date of each patient’s first pharmacy claim for either UMEC/VI or TIO during the identification period was used as their index date. Patient characteristics were assessed during the pre-index period, which covered the 12-month period before the index date. Study outcomes were evaluated during the 12-month post-index period, censored at the end of eligibility, end of data availability, or death (whichever occurred earliest). For analyses of COPD exacerbations and medical costs, the on-treatment period spanned from the index date until the earliest of discontinuation of the index medication (defined as a ≥ 45-day gap [115-day gap for mail orders] in days of supply between the end of a dispensing and the next fill, or between the end of the last dispensing and the end of data availability), a switch to a non-index medication, end of eligibility, end of data availability, or death (whichever occurred earliest). Each patient was included in one of two mutually exclusive cohorts based on whether they were receiving UMEC/VI or TIO at index.

### Patients

Patients were eligible for inclusion in the study if they had ≥ 1 pharmacy claim for fixed-dose UMEC/VI or TIO during the identification period, and had continuous enrollment with medical and pharmacy coverage during the 12-month pre- and post-index periods. Additional inclusion criteria were ≥ 1 medical claim with a COPD diagnosis code (International Classification of Diseases 9th Edition [ICD-9; before October 1, 2015] or 10th Edition [ICD-10; on or after October 1, 2015]; Additional file 1: Table 1) in any position on the index date or during the pre-index period, and age ≥ 40 years as of the index date. Patients with a moderate or severe COPD-related exacerbation on the index date were excluded, whereas patients with exacerbations at any other time during the pre-index period remained eligible. In addition, patients who had any of the following were excluded from the study: a medical claim with an ICD-9 or ICD-10 diagnosis code for asthma (Additional file 1: Table 1) during the eligibility period; a pharmacy claim for any maintenance medication containing an inhaled corticosteroid, LABA, or LAMA during the pre-index period; a pharmacy claim for a non-index maintenance medication (including single or multiple inhaler triple therapy) on the index date; or a pharmacy claim for both UMEC/VI and TIO on the index date.

### Outcomes

Patient demographics, clinical characteristics, respiratory medication history, COPD-related exacerbations, and COPD-related HCRU and medical costs were assessed during the 12-month pre-index period and on the index date.

On-treatment Kaplan–Meier (KM) rates of time-to-first (TTF) exacerbation and rates of overall, moderate, and severe COPD-related exacerbations were reported for the 12-month post-index period. Exacerbations with a start date on or before the index date were considered part of the pre-index period and were not included in the analysis of post-index outcomes. Moderate COPD-related exacerbations were outpatient or emergency room (ER) visits with a diagnosis code for COPD in the primary position (Additional file 1: Table 1) and ≥ 1 dispensing/administration of systemic corticosteroids or guideline-recommended antibiotics in the 5 days prior to or following the visit. Severe COPD-related exacerbations were defined as an inpatient hospitalization with a diagnosis code for COPD in the primary position (Additional file 1: Table 1). If a patient had ≥ 2 exacerbations within 14 days, these were considered as one exacerbation and categorized based on the most severe event.

Medication adherence measures included the mean proportion of days covered (PDC) and the proportion of adherent patients (PDC ≥ 0.8). PDC was calculated as the number of days a patient had available index medication, identified using filled prescriptions, divided by the number of days between the index date and the end of the 12-month post-index period. For patients who refilled a medication before their previous fill ran out (ie, overlapping dispensings), the refill date was considered to be the end of the previous prescription’s days of supply.

Mean COPD-related medical costs were identified as any claim with a primary or secondary diagnosis of COPD (Additional file 1: Table S1) and evaluated during the on-treatment period. Total COPD-related medical costs were stratified by hospitalization, ER visits, outpatient visits, and other visit costs.

### Data analyses

Patients were matched 1:1 between the UMEC/VI and TIO cohorts using propensity scores (PS) estimated from a multivariable logistic regression model, as previously described [14]. Patients were also exact-matched on pre-index COPD-related moderate or severe exacerbations.

Patient characteristics were evaluated during the 12-month pre-index period and compared between the UMEC/VI and TIO cohorts. Descriptive statistics including mean, standard deviation (SD), and median values for continuous variables, and relative frequencies and proportions for categorical variables were reported for pre-index characteristics in both the unmatched and matched cohorts. Standardized differences between cohorts of < 10% were not considered statistically relevant.

TTF on-treatment COPD-related exacerbation was evaluated using KM survival analysis, with KM exacerbation rates reported at 3, 6, 9, and 12 months post-index and compared between matched cohorts using hazard ratios (HR) from Cox proportional hazards regression with robust standard errors.

Rates of on-treatment COPD-related moderate and severe exacerbations were reported per 100 person-days and compared between matched cohorts with rate ratios estimated using generalized estimating equations Poisson regression. The 95% confidence intervals (CIs) and p-values were generated from non-parametric bootstrap procedures.

Mean PDC and the proportion of adherent patients (PDC ≥ 0.8) were compared between matched cohorts using generalized estimating equations and conditional logistic regression models, respectively.

On-treatment COPD-related medical costs were calculated per patient per month (PPPM), and inflation-adjusted to 2019 $US based on the medical care component of the Consumer Price Index. Statistical significance of cost differences between matched cohorts was evaluated using non-parametric bootstrap procedures.

## Results

### Study sample

**Fig. 2:**
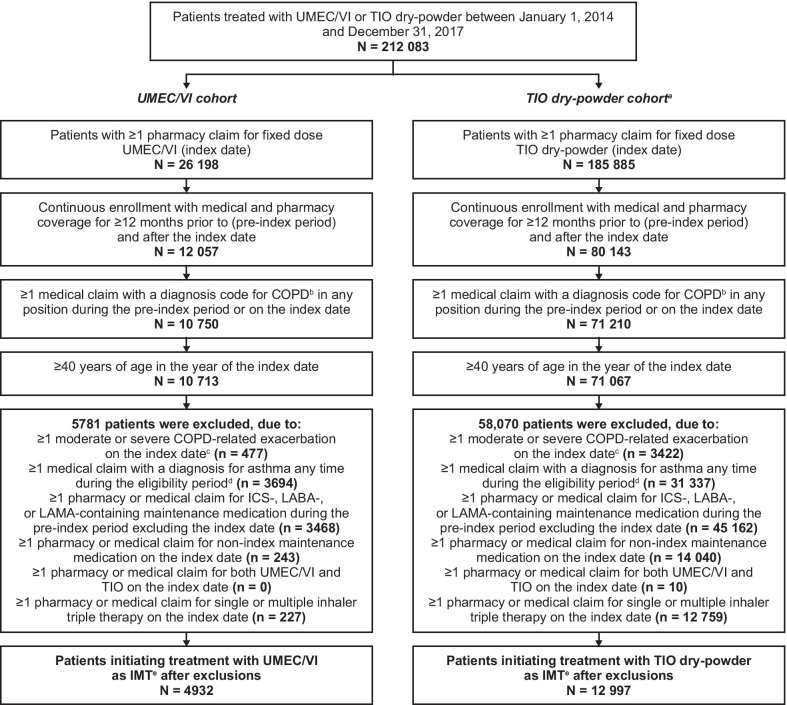
Patient disposition. ^a^Patients initiating treatment with both UMEC/VI and TIO on the index date were classified as TIO and subsequently excluded (N = 42); ^b^ICD-9/10-CM diagnostic codes for COPD are shown in Additional file 1: Table S1; ^c^Moderate COPD-related exacerbations were defined as an outpatient or ER visit with a diagnosis code for COPD in the primary position and ≥ 1 dispensing/administration of a systemic corticosteroid or guideline-recommended antibiotic within 5 days before or after the visit. Severe COPD-related exacerbations were defined as an inpatient hospitalization with a diagnosis code for COPD in the primary position (Additional file 1, Table 1); ^d^ICD-9/10-CM diagnostic codes for asthma are shown in Additional file 1: Table S1; ^e^IMT was defined as no ICS-, LABA-, or LAMA-containing maintenance medication prior to the first pharmacy claim for UMEC/VI or TIO during the identification period. COPD, chronic obstructive pulmonary disease; ER, emergency room; ICD-9, International Classification of Diseases 9th Edition; ICD-10, International Classification of Diseases 10th Edition; ICS, inhaled corticosteroids; IMT, initial maintenance therapy; LABA, long-acting β_2_-agonist; LAMA, long-acting muscarinic antagonist; TIO, tiotropium; UMEC, umeclidinium; VI, vilanterol

We identified 212 083 patients who initiated treatment with UMEC/VI or TIO between January 1, 2014 and December 31, 2017. Of these, 4932 patients initiating treatment with UMEC/VI and 12 997 patients initiating treatment with TIO met the study inclusion and exclusion criteria (Fig. 2). After PS matching, each treatment cohort included 3929 patients; patients in the UMEC/VI cohort had a longer on-treatment period than those in the TIO cohort (203 vs 139 days; standardized difference: 28.3%) (Table 1). The mean age of patients was similar for UMEC/VI and TIO (70.9 vs 70.8 years), as was the proportion of females (46.9% vs 47.0%), and the Quan-Charlson Comorbidity Index (CCI) score (3.1 vs 3.0). The most common pre-index Elixhauser comorbidities for the matched UMEC/VI and TIO cohorts were chronic pulmonary disease (90.1% vs 90.3%) and hypertension (75.7% vs 75.2%). Mean pre-index overall, moderate, and severe COPD-related exacerbations were similar in the UMEC/VI and TIO cohorts (overall: 0.46 vs 0.44; moderate: 0.30 vs 0.30; severe: 0.15 vs 0.14). The most common respiratory medications used were systemic corticosteroids (44.6% vs 45.0%), which may reflect acute treatment of pre-index exacerbations, and short-acting β_2_-agonists (38.4% vs 39.1%) for both the UMEC/VI and TIO cohorts.

**Table 1:** Baseline demographics and clinical characteristics of the UMEC/VI and TIO cohorts

	**Unmatched cohorts**			**Propensity score-matched cohorts**		
	**UMEC/VI (N = 4932)**	**TIO (N = 12 997)**	**Std diff**^**a**^** (%)**	**UMEC/VI (N = 3929)**	**TIO (N = 3929)**	**Std diff**^**a**^** (%)**
Post-index eligibility period, days, mean (SD) [median]	692.1 (259.9) [629]	999.8 (419.0) [957]	88.3	729.7 (270.6) [670]	739.3 (278.2) [677]	3.5
On-treatment follow-up period, days, mean (SD) [median]	199.4 (239.1) [90]	150.1 (234.3) [60]	20.9	203.0 (249.0) [90]	139.4 (197.9) [60]	28.3
Variables included in propensity score						
Demographics^b^						
Age, years, mean (SD) [median] Female, n (%)	70.5 (9.7) [71] 2253 (45.7)	71.9 (9.4) [72] 6432 (49.5)	14.5 7.6	70.9 (9.7) [71] 1844 (46.9)	70.8 (9.5) [71] 1847 (47.0)	0.4 0.2
Year of index date, n (%)						
2014 2015 2016 2017	77 (1.6) 639 (13.0) 1349 (27.4) 2867 (58.1)	4568 (35.1) 3485 (26.8) 2476 (19.1) 2468 (19.0)	86.8 34.7 19.7 80.4	77 (2.0) 639 (16.3) 1222 (31.1) 1991 (50.7)	81 (2.1) 642 (16.3) 1269 (32.3) 1937 (49.3)	0.7 0.2 2.6 2.7
Region,^b^ n (%)						
South West Midwest Northeast Unknown	2503 (50.8) 945 (19.2) 1007 (20.4) 466 (9.4) 11 (0.2)	4508 (34.7) 4127 (31.8) 2863 (22.0) 1456 (11.2) 43 (0.3)	32.5 28.9 3.9 5.8 2.1	1786 (45.5) 870 (22.1) 876 (22.3) 390 (9.9) 7 (0.2)	1746 (44.4) 892 (22.7) 892 (22.7) 386 (9.8) 13 (0.3)	2.0 1.3 1.0 0.3 3.0
Insurance plan type,^b^ n (%)						
Medicare Commercial	3905 (79.2) 1027 (20.8)	10 983 (84.5) 2014 (15.5)	13.8 13.8	3183 (81.0) 746 (19.0)	3183 (81.0) 746 (19.0)	0.0 0.0
Quan-CCI,^c^ mean (SD) [median]	3.0 (2.2) [3]	3.1 (2.3) [3]	1.8	3.1 (2.3) [2]	3.0 (2.3) [2]	1.8
COPD-related exacerbations^c^						
Number of exacerbations, mean (SD) [median]						
Overall Moderate Severe	0.46 (0.78) [0] 0.33 (0.68) [0] 0.13 (0.38 [0]	0.43 (0.77) [0] 0.24 (0.58) [0] 0.20 (0.47) [0]	3.8 15.6 16.2	0.46 (0.78) [0] 0.30 (0.65) [0] 0.15 (0.41) [0]	0.44 (0.78) [0] 0.30 (0.66) [0] 0.14 (0.38) [0]	1.4 0.7 1.6
Patients with exacerbations, n (%)						
Overall Moderate Severe	1640 (33.3) 1077 (21.8) 563 (11.4)	4151 (31.9) 1915 (14.7) 2236 (17.2)	2.8 18.4 16.5	1282 (32.6) 754 (19.2) 528 (13.4)	1282 (32.6) 754 (19.2) 528 (13.4)	0.0 0.0 0.0
Respiratory medications,^c^ n (%)						
Systemic corticosteroids SABA SAMA/SABA Montelukast SAMA Methylxanthines Chronic antibiotic (≥ 6 months of continuous use) N-acetylcysteine PDE-4 inhibitor	2325 (47.1) 2026 (41.1) 480 (9.7) 235 (4.8) 79 (1.6) 28 (0.6) 11 (0.2) 5 (0.1) 3 (0.1)	5036 (38.7) 4244 (32.7) 1086 (8.4) 377 (2.9) 245 (1.9) 51 (0.4) 26 (0.2) 11 (0.1) 18 (0.1)	17.0 17.5 4.8 9.7 2.2 2.5 0.5 0.5 2.5	1754 (44.6) 1508 (38.4) 371 (9.4) 181 (4.6) 68 (1.7) 19 (0.5) 8 (0.2) 5 (0.1) 1 (0.0)	1770 (45.0) 1536 (39.1) 365 (9.3) 152 (3.9) 70 (1.8) 16 (0.4) 10 (0.3) 3 (0.1) 4 (0.1)	0.8 1.5 0.5 3.7 0.4 1.1 1.1 1.6 3.0
COPD-related HCRU,^c^ mean (SD) [median]						
Hospitalizations ER visits Outpatient visits Other visits	0.21 (0.54) [0] 0.24 (0.85) [0] 3.0 (5.8) [2] 1.5 (4.1) [0]	0.34 (0.72) [0] 0.27 (0.96) [0] 2.3 (6.1) [1] 1.9 (5.0) [0]	21.1 3.6 10.7 9.4	0.25 (0.59) [0] 0.25 (0.91) [0] 2.9 (5.6) [1] 1.5 (4.2) [0]	0.24 (0.52) [0] 0.26 (0.82) [0] 2.7 (6.9) [1] 1.6 (4.4) [0]	2.4 0.5 2.8 2.2
COPD-related medical costs,^c^ $US 2019, mean (SD)						
Total medical costs Hospitalizations ER visits Outpatient visits Other visits	8167 (23 213) 4650 (19 645) 1113 (5307) 2130 (7809) 274 (1337)	11 458 (29 315) 7839 (24 071) 1546 (9618) 1720 (9639) 353 (1461)	12.4 14.5 5.6 4.7 5.6	9042 (25 234) 5552 (21 675) 1201 (5737) 2006 (7594) 284 (1412)	8642 (20 558) 5230 (16 708) 1187 (6207) 1910 (8931) 315 (1838)	1.7 1.7 0.2 1.2 1.9
Elixhauser comorbidities n (%)						
Chronic pulmonary disease Hypertension	4522 (91.7) 3733 (75.7)	11 417 (87.8) 9664 (74.4)	12.7 3.1	3539 (90.1) 2974 (75.7)	3548 (90.3) 2974 (75.2)	0.8 1.1

^a^For continuous variables, the standardized difference was calculated by dividing the absolute difference in means of the control and the case by the pooled standard deviation of both cohorts. The pooled standard deviation is the square root of the average of the squared standard deviations; for dichotomous variables, the standardized difference is calculated using the following equation where P is the respective proportion of patients in each cohort: |(Pcase-Pcontrol)| / √[(Pcase(1-Pcase) + Pcontrol(1-Pcontrol))/2]; ^b^Evaluated at the index date; ^c^Evaluated during the 12-month pre-index period

CCI, Charlson Comorbidity Index; COPD, chronic obstructive pulmonary disease; ER, emergency room; HCRU, healthcare resource utilization; PDE-4, phosphodiesterase type 4 inhibitor; SABA, short-acting β_2_-agonist; SAMA, short-acting muscarinic antagonist; SD, standard deviation; Std diff, standardized difference; TIO, tiotropium; UMEC, umeclidinium; VI, vilanterol

### On-treatment COPD-related exacerbations

KM rates for TTF on-treatment overall, moderate, and severe COPD-related exacerbation at 12 months post-index were similar between matched cohorts; differences did not reach statistical significance (HR [95% CI]; overall: 0.93 [0.82, 1.05], moderate: 0.92 [0.80, 1.06], and severe: 1.07 [0.86, 1.33]; Additional file 1: Fig. 1). At 3 months post-index, more patients receiving TIO compared with patients receiving UMEC/VI had experienced a COPD-related overall (n = 313 vs n = 295) or moderate (n = 224 vs n = 212) exacerbation, although the difference in exacerbation risk did not reach statistical significance. The rates of on-treatment exacerbations per 100 person-days were similar between patients initiating treatment with UMEC/VI (overall: 0.12; moderate: 0.08; severe: 0.04) and patients initiating treatment with TIO (overall: 0.12; moderate: 0.09; severe: 0.03), with no statistically significant differences in rate ratios observed between cohorts (Table 2).

**Table 2:** Rate of on-treatment exacerbations for the UMEC/VI and TIO cohorts

COPD-related exacerbation outcomes	Number of events		Rate (per 100 person-days)		Rate ratio (95% CI)	*p* value
	UMEC/VI (N = 3929)	TIO (N = 3929)	UMEC/VI (N = 3929)	TIO (N = 3929)		
On-treatment period, mean (SD) [median]	150.1 (135.5) [90]	111.2 (116.6) [60]	–	-	–	–
Total person-days	589 596	436 824	–	–	–	–
Exacerbations						
Overall	698	541	0.12	0.12	0.95 (0.84, 1.07)	0.473
Moderate	476	392	0.08	0.09	0.90 (0.77, 1.06)	0.152
Severe	222	149	0.04	0.03	1.10 (0.85, 1.35)	0.477

CI, confidence interval; COPD, chronic obstructive pulmonary disease; SD, standard deviation; TIO, tiotropium; UMEC, umeclidinium; VI, vilanterol

### Medication adherence

Overall, adherence was significantly higher for the UMEC/VI cohort compared with the TIO cohort for both mean PDC (0.44 vs 0.37; *p* < 0.001) and the proportion of patients achieving PDC ≥ 0.8 (22.0% vs 16.4%; *p* < 0.001) (Table 3). Patients initiating treatment with UMEC/VI were 44% more likely to achieve PDC ≥ 0.8 compared with patients initiating treatment with TIO (odds ratio [95% CI]: 1.44 [1.28, 1.61]).

**Table 3:** Medication adherence for the UMEC/VI and TIO cohorts

	UMEC/VI (N = 3929)	TIO (N = 3929)	*p* value^a^	Difference (95% CI)	Odds ratio (95% CI)
PDC, mean (SD) [median]	0.44 (0.32) [0.33]	0.37 (0.31) [0.25]	< 0.001	0.06 (0.05, 0.08)	–
Proportion with PDC ≥ 0.8, n (%)	863 (22.0)	646 (16.4)	< 0.001	–	1.44 (1.28, 1.61)

^a^*p*-values were calculated using paired *t*-tests for mean PDC and McNemar tests for proportion of patients with PDC ≥ 0.8

CI, confidence interval; PDC, proportion of days covered; SD, standard deviation; TIO, tiotropium; UMEC, umeclidinium; VI, vilanterol

### On-treatment COPD-related medical costs

Patients initiating treatment with UMEC/VI had a significantly lower mean on-treatment total medical costs PPPM compared with patients initiating treatment with TIO ($867 vs $1095; mean difference: $228; *p* = 0.028; Table 4). The difference in total medical costs was primarily driven by significantly lower outpatient visit costs ($296 vs $462; mean difference: $166; p = 0.036), which accounted for 73% of the total medical cost difference.

**Table 4:** On-treatment COPD-related medical costs for the UMEC/VI and TIO cohorts

	UMEC/VI (N = 3929)	TIO (N = 3929)	Cost difference (95% CI)	*p* value
On-treatment period, mean days (SD) [median]	203.0 (249.0) [90]	139.4 (197.9) [60]	–	–
COPD-related medical costs, $US 2019 PPPM, mean (SD)				
Total medical costs	867 (3259)	1095 (7958)	− 228 (− 504, − 15)	0.028
Hospitalizations	406 (1837)	461 (2414)	− 54 (− 161, 49)	0.309
ER visits	123 (2275)	125 (707)	− 2 (− 38, 36)	0.914
Outpatient visits	296 (1129)	462 (7433)	− 166 (− 409, − 7)	0.036
Other visits	41 (181)	48 (176)	− 6 (− 15, 3)	0.184

CI, confidence interval; COPD, chronic obstructive pulmonary disease; PPPM, per patient per month; SD, standard deviation; TIO, tiotropium; UMEC, umeclidinium; VI, vilanterol

## Discussion

In this retrospective cohort study, US claims data were used to evaluate on-treatment COPD-related exacerbations and medical costs, as well as medication adherence in patients with COPD initiating maintenance therapy with UMEC/VI or TIO. KM rates of on-treatment COPD-related exacerbations were similar between patients receiving UMEC/VI and those receiving TIO. However, patients receiving UMEC/VI had a longer on-treatment period, significantly better medication adherence, and significantly lower total COPD-related medical costs than patients receiving TIO, highlighting the clinically relevant benefits of initiating maintenance therapy with UMEC/VI over TIO in a real-world population of patients with COPD.

Adherence to COPD medications has been shown to be suboptimal and generally lower than among patients with other long-term conditions, such as coronary artery disease and diabetes [15, 16]. One study that assessed adherence to COPD maintenance medication reported a mean PDC of 0.47 with only 20.8% of patients achieving adherence based on PDC ≥ 0.8 [16]. Poor medication adherence among patients with COPD is associated with more frequent hospital admissions, increased mortality, and higher healthcare costs than for patients who are more adherent to their COPD treatment [7, 8]. In the current study, both mean PDC and the proportion of patients achieving a clinically relevant level of adherence (PDC ≥ 80%) were significantly higher in patients treated with UMEC/VI than among those treated with TIO; however, no significant differences in moderate and severe exacerbations were detected between cohorts, possibly due to the low incidence of post-index exacerbations observed in the study population. These data complement previous findings of improved adherence among patients treated with UMEC/VI versus other combination treatments including budesonide/formoterol [17], tiotropium/olodaterol [18], and fluticasone propionate/salmeterol [19]. Reasons for better adherence to UMEC/VI compared with TIO were not evaluated in the current study; however, previous research has shown that a number of factors can contribute to adherence rates, including patients’ characteristics or views regarding the treatment of their disease, complexity of the treatment regimen, and the inhaler device [20–22]. To account for differences in pre-index characteristics between cohorts, patients were PS matched on observed measures; however, unobservable measures, including views regarding the treatment, may have differed. Complexity of the treatment regimen was unlikely to have contributed to medication adherence in this study since both UMEC/VI and TIO are once-daily single dry-powder inhaler medications. However, UMEC/VI and TIO are administered using different inhaler devices (ELLIPTA and HandiHaler, respectively), which may have contributed to the improved adherence to UMEC/VI seen in the present study. Indeed, evidence suggests that patients prefer the ELLIPTA inhaler compared with the HandiHaler [23].

Better treatment adherence has been linked to lower medical costs in patients with COPD, which may be attributed to reduced HCRU [10]. In the current study, mean on-treatment total medical costs PPPM were $228 lower for patients initiating treatment with UMEC/VI compared with TIO, which may in part be attributable to better adherence to UMEC/VI. Our findings support those of several economic modeling analyses from the US, UK, and Spain, which have found that UMEC/VI may be considered a more cost-effective treatment compared with TIO [13, 24–26]. Together, these data provide important insights for payers in a growing market of LAMAs and LAMA/LABA combination therapies for the treatment of COPD.

Notably, the lower total medical costs observed among patients receiving UMEC/VI compared with TIO in this study were primarily driven by lower outpatient visit costs, which are closely linked to the definition of a moderate COPD-related exacerbation. These findings may suggest that the lower total costs are reflective of the trend toward a lower risk of moderate exacerbations seen among patients initiating treatment with UMEC/VI. Moreover, fewer patients who initiated treatment with UMEC/VI experienced an on-treatment moderate exacerbation at 3 months compared with patients initiating treatment with TIO. Although statistical significance was not reached for KM rates of exacerbations, this raises the possibility that patients receiving TIO may have a greater need for pharmacologic treatment of acute exacerbations.

There are several strengths of this study. The study population was extracted from a large database representing a geographically diverse sample of the US population and provides real-world evidence regarding the use of UMEC/VI and TIO in patients with COPD. In addition, any confounding effects of patient characteristics on study outcomes were minimized through PS matching. However, certain limitations of the study should also be considered, many of which are inherent to claims-based studies. First, medical and pharmacy claims provide only indirect measures of exacerbations and adherence. A pharmacy claim for a dispensed medication does not necessarily indicate that the patient used the medication as prescribed. In addition, over-the-counter or sample medications and treatments received in hospital care would not have been captured in the claims data. These factors may have affected the estimation of adherence in the current study; however, they are likely to have been equally applicable to both treatment cohorts. Second, given the low overall rate of COPD-related exacerbations in the study population, a statistically significant difference in exacerbations between the two treatment cohorts may have been difficult to detect. Also, patients who may have experienced severe COPD-related exacerbations and died before completing 12 months of continuous enrollment were excluded from the study, which may have further contributed to the relatively mild COPD population with respect to exacerbations experienced during the study period. Third, although the treatment cohorts were PS matched to minimize confounding effects, there remains the possibility of confounding due to unmeasured variables, such as symptoms. Finally, there is limited generalizability of the results to the uninsured US population, non-US populations, or patients using alternative TIO formulations such as TIO administered with a soft mist inhaler. Nonetheless, these data provide valuable insights into the comparative effects of UMEC/VI and TIO in a real-world population of patients with COPD from the USA.

## Conclusions

In this retrospective US claims database study, patients with COPD had low overall on-treatment exacerbation rates. The findings show that compared with TIO, the use of UMEC/VI as IMT may reduce medical costs, possibly through increased adherence to therapy. This provides valuable information for physicians considering IMT options for patients with COPD.

## Supplementary Information

**Additional file 1**. **Supplementary Table 1** Supplemental table showing the ICD-9/10-CM diagnostic codes for COPD, COPD-related exacerbations and asthma. **Supplementary Fig. 1** Supplemental figure showing the Kaplan–Meier rates of (A) moderate and (B) severe exacerbations for the UMEC and TIO cohorts.

## Data Availability

Information on GlaxoSmithKline’s (GSK) data sharing commitments and requesting access to anonymized individual participant data and associated documents from GSK-sponsored studies can be found at www.clinicalstudydatarequest.com. The data reported in this publication are contained in a database owned by Optum and contain proprietary elements. Therefore, it cannot be broadly disclosed or made publicly available at this time. The disclosure of this data to third-party clients assumes certain data security and privacy protocols are in place and that the third-party client has executed Optum’s standard license agreement which includes restrictive covenants governing the use of the data.

## Abbreviations

CCI: Charlson Comorbidity Index
CI: Confidence interval
COPD: Chronic obstructive pulmonary disease
ER: Emergency room
GSK: GlaxoSmithKline
HCRU: Healthcare resource utilization
HR: Hazard ratio
HIPAA: Health Insurance Portability and Accountability Act of 1996
ICD-9/10-CM: International Classification of Diseases 9th/10th Edition Clinical Modification
IMT: Initial maintenance therapy
KM: Kaplan–Meier
LABA: Long-acting β_2_-agonist
LABD: Long-acting bronchodilator
LAMA: Long-acting muscarinic antagonist
PDC: Proportion of days covered
PPPM: Per patient per month
PS: Propensity score
SAMA: Short-acting muscarinic antagonist
SD: Standard deviation
TIO: Tiotropium
TTF: Time-to-first
UMEC: Umeclidinium
VI: Vilanterol
