# Supplementary material for: Time-to-first exacerbation, adherence, and medical costs among US patients receiving umeclidinium/vilanterol or tiotropium as initial maintenance therapy for chronic obstructive pulmonary disease: a retrospective cohort study
Source: BMC Pulm Med. 2021 Jul 31;21:253. doi: 10.1186/s12890-021-01612-5 (PMC8325860; doi:10.1186/s12890-021-01612-5)
Supplement: Supplementary file 1 — Additional file 1. Supplementary Table 1 Supplemental table showing the ICD-9/10-CM diagnostic codes for COPD, COPD-related exacerbations and asthma. Supplementary Fig. 1 Supplemental figure showing the Kaplan–Meier rates of (A) moderate and (B) severe exacerbations for the UMEC and TIO cohorts. [file 12890_2021_1612_MOESM1_ESM.pdf]

Additional File 1

Plain language summary

Patients with chronic obstructive pulmonary disease (COPD) are often prescribed daily long-acting maintenance medications to help manage their disease. These medications include long-acting bronchodilators, which relax the lung muscles and open the airways. The main types of long-acting bronchodilators are long-acting muscarinic antagonists (LAMAs) and long-acting β_2-agonists (LABAs), which may be used alone or in combination (LAMA/LABA). Treatment adherence measures whether patients take their medication as prescribed by their doctors. Good adherence to maintenance treatment is important for managing the symptoms of COPD, including exacerbations (flare-ups of disease), and is also linked to lower healthcare costs.

This study used data from a United States health insurer claims database to compare exacerbations, adherence and medical costs among eligible patients with COPD who initiated treatment with either umeclidinium/vilanterol (UMEC/VI), a LAMA/LABA combination treatment, or tiotropium (TIO), a LAMA treatment. This study found that the risk of exacerbations while patients were receiving treatment was similar between patients in both treatment groups. However, patients receiving UMEC/VI had better adherence to treatment and lower healthcare costs while receiving their treatment compared with patients receiving TIO.

These findings provide valuable information about the risk of exacerbations, medication adherence, and medical costs in patients with COPD receiving maintenance treatment with UMEC/VI or TIO. This information may be useful for physicians and healthcare providers who are considering which initial maintenance therapy is most appropriate to prescribe for patients with COPD.

Supplementary Table 1. ICD-9/10-CM diagnostic codes for COPD, COPD-related exacerbations and asthma

Code	Description	COPD	COPD-related exacerbation	Asthma
**ICD-9-CM**				
466	Acute bronchitis		✓	
490	Bronchitis, not specified as acute or chronic		✓	
491	Chronic bronchitis	✓	✓	
491	Simple chronic bronchitis	✓	✓	
491.1	Mucopurulent chronic bronchitis	✓	✓	
491.2	Obstructive chronic bronchitis, without exacerbation	✓	✓	
491.21	Obstructive chronic bronchitis, with (acute) exacerbation	✓	✓	
491.22	Obstructive chronic bronchitis with acute bronchitis	✓	✓	
491.8	Other chronic bronchitis	✓	✓	
491.9	Unspecified chronic bronchitis	✓	✓	
492	Emphysema	✓	✓	
492	Emphysematous bleb	✓	✓	
492.8	Other emphysema	✓	✓	
493	Extrinsic asthma, unspecified		✓	✓
493.01	Extrinsic asthma, with status asthmaticus		✓	✓
493.02	Extrinsic asthma, with (acute) exacerbation		✓	✓
493.11	Intrinsic asthma, with status asthmaticus		✓	✓

493.12	Intrinsic asthma, with (acute) exacerbation		✓	✓
493.21	Chronic obstructive asthma with status asthmaticus		✓	✓
493.22	Chronic obstructive asthma, with (acute) exacerbation		✓	✓
493.9	Asthma, unspecified, unspecified status		✓	✓
493.91	Asthma, unspecified, with status asthmaticus		✓	✓
493.92	Asthma, unspecified, with (acute) exacerbation		✓	✓
494.1	Bronchiectasis with acute exacerbation		✓	
496	Chronic airway obstruction, not elsewhere classified	✓	✓	
518.81	Acute respiratory failure		✓	
518.82	Other pulmonary insufficiency, not elsewhere classified		✓	
518.84	Acute and chronic respiratory failure		✓	
799.1	Respiratory arrest		✓	
**ICD-10-CM**				
J20	Acute bronchitis		✓	
J200	Acute bronchitis due to Mycoplasma pneumoniae		✓	
J201	Acute bronchitis due to Hemophilus influenzae		✓	
J202	Acute bronchitis due to streptococcus		✓	
J203	Acute bronchitis due to coxsackievirus		✓	

J204	Acute bronchitis due to parainfluenza virus		✓
J205	Acute bronchitis due to respiratory syncytial virus		✓
J206	Acute bronchitis due to rhinovirus		✓
J207	Acute bronchitis due to echovirus		✓
J208	Acute bronchitis due to other specified organisms		✓
J209	Acute bronchitis, unspecified		✓
J40	Bronchitis, not specified as acute or chronic		✓
J41	Simple and mucopurulent chronic bronchitis	✓	✓
J410	Simple chronic bronchitis	✓	✓
J411	Mucopurulent chronic bronchitis	✓	✓
J418	Mixed simple and mucopurulent chronic bronchitis	✓	✓
J42	Unspecified chronic bronchitis	✓	✓
J43	Emphysema	✓	✓
J430	Unilateral pulmonary emphysema [MacLeod's syndrome]	✓	✓
J431	Panlobular emphysema	✓	✓
J432	Centrilobular emphysema	✓	✓
J438	Other emphysema	✓	✓
J439	Emphysema, unspecified	✓	✓
J44	Other chronic obstructive pulmonary disease	✓	✓
J440	Chronic obstructive pulmonary disease with acute lower respiratory infection	✓	✓

J441	Chronic obstructive pulmonary disease with (acute) exacerbation	✓	✓	
J449	Chronic obstructive pulmonary disease, unspecified	✓	✓	
J4520	Mild intermittent asthma, uncomplicated		✓	
J4521	Mild intermittent asthma with (acute) exacerbation		✓	
J4522	Mild intermittent asthma with status asthmaticus		✓	
J4531	Mild persistent asthma with (acute) exacerbation		✓	✓
J4532	Mild persistent asthma with status asthmaticus		✓	✓
J4541	Moderate persistent asthma with (acute) exacerbation		✓	✓
J4542	Moderate persistent asthma with status asthmaticus		✓	✓
J4551	Severe persistent asthma with (acute) exacerbation		✓	✓
J4552	Severe persistent asthma with status asthmaticus		✓	✓
J45901	Unspecified asthma with (acute) exacerbation			✓
J45902	Unspecified asthma with status asthmaticus		✓	✓
J45909	Unspecified asthma, uncomplicated		✓	✓
J45998	Other asthma		✓	✓

J471	Bronchiectasis with (acute) exacerbation		✓
J80	Acute respiratory distress syndrome		✓
J960	Acute respiratory failure		✓
J9600	Acute respiratory failure, unspecified whether with hypoxia or hypercapnia		✓
J9601	Acute respiratory failure with hypoxia		✓
J9602	Acute respiratory failure with hypercapnia		✓
J962	Acute and chronic respiratory failure		✓
J9620	Acute and chronic respiratory failure, unspecified whether with hypoxia or hypercapnia		✓
J9621	Acute and chronic respiratory failure with hypoxia		✓
J9622	Acute and chronic respiratory failure with hypercapnia		✓
J969	Respiratory failure, unspecified		✓
J9690	Respiratory failure, unspecified, unspecified whether with hypoxia or hypercapnia		✓
J9691	Respiratory failure, unspecified with hypoxia		✓
J9692	Respiratory failure, unspecified with hypercapnia		✓
R092	Respiratory arrest		✓

COPD, chronic obstructive pulmonary disease; ICD-9/10-CM, International Classification of Diseases, $9^{th}/10^{th}$ Revision, Clinical Modification.

Supplementary Figure 1. Kaplan–Meier rates of (A) moderate and (B) severe exacerbations for the UMEC and TIO cohorts

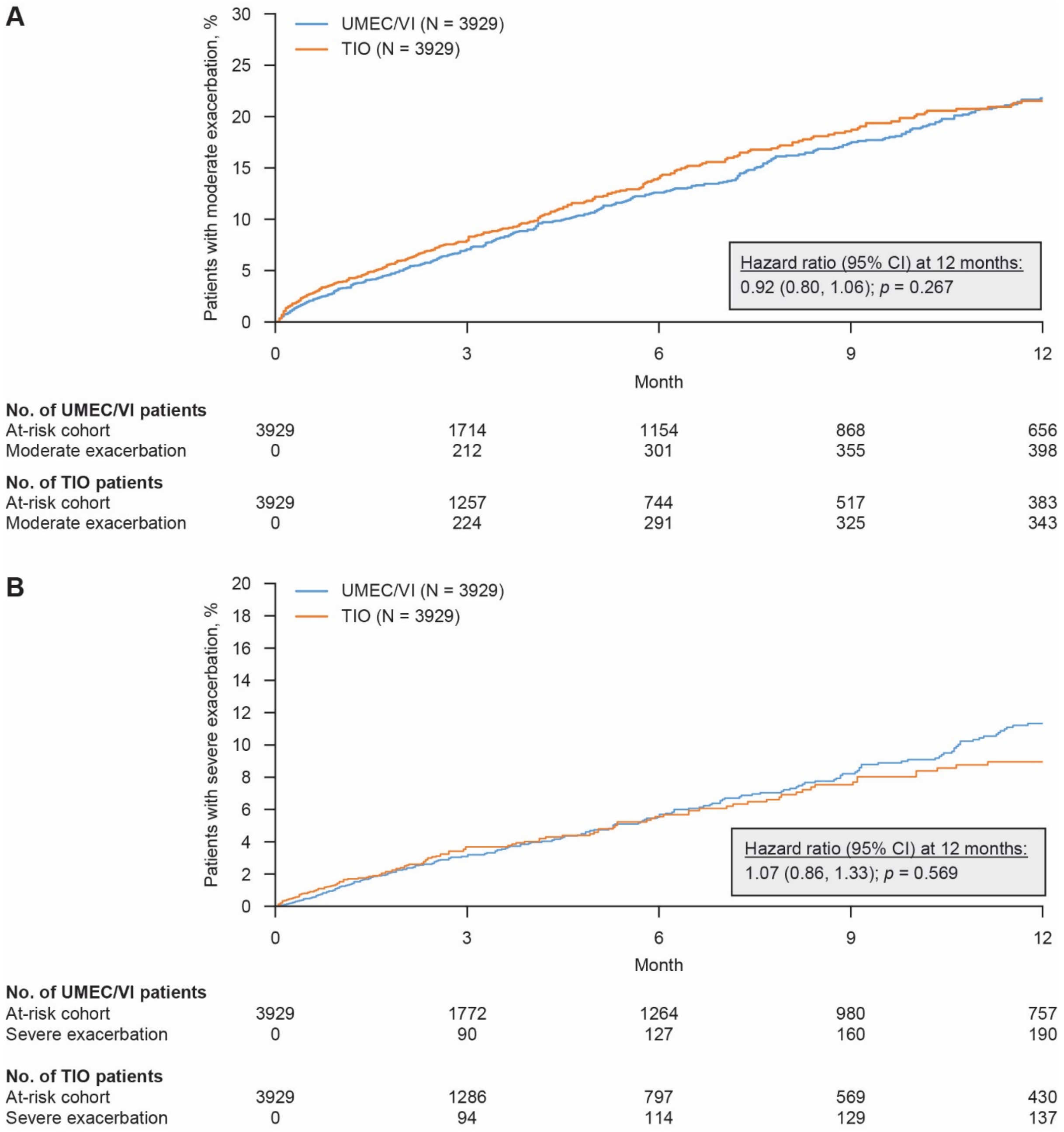

CI, confidence interval; TIO, tiotropium; UMEC, umeclidinium/VI, vilanterol.
